# Adverse effects of maternal enterovirus infection on the pregnancy outcome: a prospective and retrospective pilot study

**DOI:** 10.1186/s12985-018-0978-7

**Published:** 2018-04-16

**Authors:** Z. Khediri, C. Vauloup-Fellous, A. Benachi, J. M. Ayoubi, L. Mandelbrot, O. Picone

**Affiliations:** 1 Service de gynécologie et obstétrique and Risk in pregnancy university department, Hôpital Louis-Mourier, Assistance publique-Hôpitaux de Paris, 178, rue des Renouillers, 92700 Colombes, France; 2 Université Paris-Diderot, 75013 Paris, France; 3 Inserm U1193, virologie, WHO Rubella NRL, National Reference Laboratory for Maternofetal Rubella Infections, AP-HP, hôpital Paul-Brousse, groupe hospitalier universitaire Paris-Sud, université Paris-Sud, 94804 Villejuif, France; 4 Department of Obstetrics, Gynecology and Reproductive Medicine and Centre, maladies rares : hernie de coupole diaphragmatique, hôpital Antoine-Béclère, AP-HP, université Paris Sud, 157, rue de la Porte-de-Trivaux, 92140 Clamart, France; 5 Department of obstetrics and gynecology, hôpital Foch, 92120 Suresnes, France; 6 EA2493, UFR des sciences de la santé Simone-Veil, université Versailles Saint-Quentin-en-Yvelines, 78180 Montigny-le-Bretonneux, France; 7 IAME (Infection, Antimicrobials, Modelling, Evolution), INSERM, UMR 1137 – UFR de Médecine Paris 7 Denis Diderot, 16 rue Henri Huchard, B.P. 416 – 75870 Paris cedex 18, France; 8 Groupe de Recherche sur les Infections Pendant la Grossesse (GRIG), Vélizy, France

## Abstract

**Background:**

Enteroviruses account for about one billion infections worldwide each year, the majority remain asymptomatic. Data on enterovirus infections during pregnancy appear to be very rare. Several cases have been reported in the literature of fetal and neonatal complications attributed to these viruses, but prospective data on these infections during pregnancy are not available.

**Objective:**

To estimate the prevalence of enterovirus infections in febrile syndromes in pregnant women, and in case of in utero fetal death (IUFD).

**Methods:**

Ttri-centric observational cohort study. We performed prospective inclusion for patients with fever during a four-month period. We also analyzed the amniotic fluid in patients with unexplained IUFD retrospectively during a five-year period. Investigations of enteroviruses are made by RT-PCR from routine biological samples (amniocentesis, RT-PCR in maternal blood or CSF).

**Results:**

Prospectively, 33 patients were included during the study period. We have identified 4 cases of confirmed enterovirus infection (12.4%). We have recorded a severe form of perinatal enterovirus infection involving the vital prognosis of the newborn.

In the retrospective cohort of 75 IUFD cases, we had only one case of enterovirus-positive RT-PCR in amniotic fluid during 5 years, meaning a frequency of 1.3%. We did not had any positive EV case in case of early miscarriage, but the limited number of inclusions cannot help us to conclude.

**Conclusion:**

Enteroviruses are probably an underestimated cause of obstetric and neonatal complications. Investigation of enterovirus by PCR should be discussed during pregnancy and peripartum in case of febrile syndrome with no obvious bacterial cause, and unexplained IUFD.

## Introduction

Enterovirus (EV) infections are responsible for about one billion infections each year worldwide, of which majority is asymptomatic (90%) [1, 2]. The enteroviruses have a ubiquitous distribution. The spread of EV can be sporadic, endemic, epidemic, and even pandemic. Several serotypes can co-circulate during the same period and within the same population. They can lead to variable clinical presentations ranging from isolated fever to severe sepsis or multi-visceral failure. Usual transmission is the by the fecal-oral route. Infection can be transmitted through the consumption of contaminated water, or directly by contaminated people. Respiratory transmission may occur by droplets or by direct contact of the contaminated hand with the mouth, nose or eyes [3].

However, these infections during pregnancy have been little studied in the literature. A few cases of adverse obstetrical outcome caused by these viruses (miscarriage and fetal death) [1] have been reported. In our previous experience, we managed two cases of enterovirus infection in pregnant women with severe obstetrical consequences (hydrops, myocarditis and fetal death) [3, 4]. Adverse outcomes of EV infections are mostly known to neonatologists, since many cases have been published of early neonatal enteroviral myocarditis, severe meningoencephalitis or even death [5, 6]. There are currently no data available regarding the maternal-fetal transmission rate and no recommendations for the diagnosis, screening or management of these infections during pregnancy.

## Objectives

We evaluated the prevalence and epidemiological characteristics of enterovirus infection among pregnant women with unexplained fever and/or early miscarriage, as well as among women having IUFD of unknown etiology.

## Study design

This was a prospective and retrospective, multicenter, cohort study, conducted in three centers in the Paris region: Louis Mourier (AP-HP), Antoine Béclère (AP-HP) and Foch hospitals. Each of the three centers performs prenatal care and delivery for 3000–3500 pregnant women yearly.

Testing for enterovirus was introduced in these institutions in January 2016 as part of the routine work-up of pregnant patients presenting in the emergency rooms (ER) for unexplained fever. Unexplained fever was defined as rectal temperature > 38 °C, without any clinical or biological evidence of chorioamniotitis (such as fetid or brown/green tainted amniotic fluid), urinary tract infection, or any other bacterial infection. In that case, testing for enterovirus infection was done immediately in maternal blood sample. As EV can cause early pregnancy loss, women presenting with an early miscarriage were offered enterovirus PCR by an anorectal swab.

Prospective study: We analyzed prospectively the results of EV testing from February to June 2016. Data collection such as pregnancy follow-up, medical history and biological tests was extracted from patient files. In case of a positive enterovirus exam, the pregnancy outcomes were collected monthly during the pregnancy and after delivery, directly in maternal and neonatal medical files, by the study coordinator. Since it was an observational study, no additional monitoring or treatment was provided for positive enterovirus cases, and patients received usual patient care.

Retrospective study: A 5-year retrospective study of all cases of unexplained IUFD for which stored amniotic fluid was available was also conducted. A total of 75 samples were eligible, stored at − 80 °C in the virology Laboratory of Paul Brousse Hospital (AP-HP). Enterovirus search in maternal blood, stool, or in amniotic fluid was based on EV real-time RT-PCRs technique (Enterovirus R-gene, bioMérieux/Argene). This technique is approved for CSF, blood, respiratory specimens and anorectal swab [7]. The clinical performance of the ENTEROVIRUS R-gene assay for amniotic fluid was evaluated by testing clinical specimens, comparatively to the routinely used diagnostic techniques (home PCR and culture) before initiating the retrospective study.

The study was approved by the institutional review board (CEERB PARIS NORD, IRB00006477, Project N°16–025).

## Results

During the 4-month period of the study, we analyzed 39 pregnant women, of whom 31 were tested for enterovirus infection because of a febrile syndrome of undetermined etiology and 8 were tested because of miscarriage before 12 gestational weeks. Seventy-five cases of IUFD of unknown etiology during a five-year period were retrospectively tested for enterovirus in the amniotic fluid samples.

The mean maternal age in the prospective group was 33.45 ± 5.34 years with a range of 19 to 43 years. 14.7% of women were primigravida and mean parity was 2.48. The prevalence of enterovirus infection in the population of febrile pregnant women was 4/31 (12.9%). None of the 8 patients presenting an early miscarriage had a positive test for EV. The epidemiological and clinical characteristics of four enterovirus infected patients are described in Table 1. Among these patients with enterovirus infection, we did not record any adverse pregnancy outcome, but there was one case of severe neonatal enterovirus infection. The patient had a confirmed enterovirus infection; she was febrile at the beginning of labor. Examination of the newborn was normal. The newborn developed viral enterovirus meningitis on day 5 of life followed by a severe cardiogenic shock following myocarditis with left ventricular dysfunction, leading to multi-visceral failure requiring transfer to the intensive care unit. Clinical improvement occurred after a 10-days stay in intensive care. EV infection was confirmed in blood RT-PCR tests in the newborn.

**Table 1:** Clinical, biological, and pregnancy characteristics, in EV+ patients

	Case 1	Case 2	Case 3	Case 4
Parity	1	2	1	0
Gestational age at diagnosis (Weeks)	35	18	38	16
Reason for consultation	Fever and contractions	Headache	Fever and contractions	Fever and headache
EV infection test	RT-PCR on maternal blood	RT-PCR on CSF	RT-PCR on maternal blood	RT-PCR on maternal blood
Clinical condition				
Temperature (°C)	39°	38,8°	38,6°	39,5°
Headache	–	Yes, intense	–	Yes
Meningitis syndrome	–	Yes	–	–
ENT signs	–	–	–	–
Digestive signs	–	–	Vomiting	–
Foot-hand-to-mouth syndrome	–	–	–	–
Skin rash	Yes: non-pruritic maculopapular	–	–	–
Uterine contractions	Yes	–	Yes	–
Biology				
CRP (mg/l)	36	21	9	18
WBC (10^3/mm3)	6,3	10,4	6,4	8,2
CBEU	–	–	–	–
Vaginal swab	–	–	–	–
Blood culture	–	–	–	–
Pregnancy outcome				
Pregnancy termination	–	–	–	–
Living birth	Yes	Yes	Yes	Yes
Gestational age at delivery (Weeks)	37 + 4d	40 + 1d	38 + 6d (Triggering, suspicious FHR)	40 + 5d
Neonatal complication	Yes, Multivisceral failure	–	–	–
Neonatal fever	yes	–	–	–
EV infection in new born	confirmed, in CSF	–	–	–

Among the 75 cases of IUFD of unknown etiology during a five-year period were tested for enterovirus in the amniotic fluid samples, 1 one was found positive for EV (1.3%) (Qualitative RT-PCR). The mean maternal age was 35.42 ± 5.01 with extremes of 24 and 47 years. The mean gestational age of the IUFD was 25 WG ± 7 with extremes of 15 and 41 WG.

In this infected case, the patient was multiparous, with no significant medical history, and IUFD occurred without any symptom of infection, such as fever, urinary tract infection or chorioamniotitis. Although fetal pathologic examination was not performed, no other cause for the fetal demise was found.

## Discussion

We demonstrated that almost 12% of pregnant women with an unexplained fever and 1% of unexplained IUFD are infected with EV.

An analysis during a longer period would be useful because climate factors influence the transmission of EV. In temperate countries, major epidemics occurring in the autumn-summer period, whereas in tropical countries, EVs can circulate throughout the year. In France, the number of EV infections starts to increase each year in summer and early autumn, with a peak in July, before gradually fading [2] but sporadic cases may occur. Our prospective part of the study took place outside the epidemic periods. Therefore, our series may underestimate the incidence and the consequences of EV infection in pregnant women.

Among pregnant women, multipara are at particular risk of infection, probably because infections are predominantly in children under 15 years of age [8] and mothers are the primary caregivers for infected young children [9]. In our study 3 patients among the 4 EV+ had young dependent children. Diagnosis is difficult in adults because the infection is mostly asymptomatic and symptoms are non-specific. EV is little known by medical teams and therefore remains underdiagnosed. The symptoms in pregnant women do not differ from those of adults in general. Clinical signs are often limited to fever, but a flu-like syndrome, diarrhea, conjunctivitis or rash (exanthema, foot-and-mouth syndrome) may also be present [2]. Clinical presentation in children and adults can be complicated by myocarditis, pericarditis, aseptic meningitis, and sometimes encephalitis [10]. One of the 4 patients in our series had confirmed laboratory meningitis by RT-PCR on a CSF lumbar puncture. Potentially fatal systemic infections occur preferentially in patients with humoral immunosuppression or immunosuppressive therapy [8]. In our series, clinical evolution was favorable in all mothers, but no patient had immunosuppression.

The positive diagnosis of enterovirus infection is biological. The most specific and sensitive examination is RT-PCR on blood, throat, CSF or amniotic fluid in search of viral RNA. Interpretation of a positive RT-PCR depends on the sampling site. Indeed, it is highly significant if positive in CSF or amniotic fluid, and on the other hand insufficient in stools because it is not specific of an acute infection [11]. Positive enterovirus in the throat is very frequent even in asymptomatic individuals. Therefore, blood is more specific in this situation. In fact, RT-PCR enterovirus is often positive on maternal blood in the acute phase of infection, usually when the patient is still febrile. It soon becomes negative but remains positive for several days (up to 3 weeks) in the stool. It is for this reason that in our prospective study, febrile patients had an EV test by RT-PCR on EDTA tube of maternal blood, whereas the patients presenting an early miscarriage without fever had to have an endo-anal swab or a stool sampling, since both are having the same sensivity.

During EV infection, the biological inflammatory syndrome is not constant [9]. In our series, the CRP assay was positive in all patients with EV infection, but the rate never exceeded 40 mg/l, and leukocytosis was not observed in any of the infected patients.

Enterovirus infection may be responsible for miscarriage if infection occurs in early pregnancy. Several mechanisms may be involved in miscarriages: inflammation of the uterus may interfere with implantation, as well as alteration of organogenesis, especially in fetal brain and heart [12]. In our prospective series, we included a total of 8 patients with early (first trimester) miscarriage, which is a very small number. We were unable to identify enterovirus infections in this subgroup of patients. The reason is obvious: several eligible patients refused endo-anal swab, which was considered more disturbing than a conventional blood test.

Many publications have also linked EV infection to unexplained stillbirth. One of the first articles to describe a case of enterovirus infection in utero with fetal demise dates from 1988, when Bryce and al. reported a Coxsackievirus infection that occurred early in pregnancy, leading to a severe IUGR in the second trimester and then to an IUFD at 30 weeks’ gestation of a non-malformed 700 g fetus [13]. Tassin and al. described a case of echovirus 11 infection in early pregnancy with transmission to the fetus. This was a bi-amniotic bi-chorionic twin pregnancy with fetal fetal demise of one twin at 14 weeks of age and the development of severe pulmonary hypoplasia at 32 weeks in the surviving twin, responsible for death on the first day of life [4]. Other recent cases of IUFD have been described at 29 weeks following an echovirus 11 infection (E-11) [14] and at 36 weeks due to Coxsackievirus A16 [15]. The diagnosis of in utero transmission of the enterovirus is most often performed by RT-PCR in the amniotic fluid either prospectively or retrospectively, or at pathology examination of placental or cerebral tissues [7, 16]. In our retrospective series, we found one positive RT-PCR on amniotic fluid out of the 75 selected samples, a prevalence of EV infection of 1.33% in unexplained IUFD. This rate is comparable to, or even higher than many other infectious causes of IUFD that are included in the common work-up, such as CMV (1.5–3.1%), or rubella [17, 18]. This result justifies a larger study of RT-PCR EV on post-IUFD amniotic fluids, in order to be able to confirm these results and implement the enterovirus infection screening in case of unexplained fetal loss.

In case of EV positive RT-PCR, and in the absence of any other cause of fetal loss, parents may be reassured regarding the risk of IUFD recurrence in future pregnancies.

Fetal enterovirus infection has been described to be responsible for sonographic findings that include cerebral ventriculomegaly, cardiomyopathy with ventricular dysfunction [19], and polyhydramnios associated with ascites, pericardial and pleural effusions which can lead to death after multi-visceral failure [20]. Bonnin and al reported a case of congenital infection with Coxsackie virus B5, manifesting at 34 weeks, by decreased fetal movements, polyhydramnios and myocarditis. Delivery was performed at 35 weeks because of severe arrhythmia. A 7-day treatment with Cordarone was necessary at birth and cardiac examination at 1 month was strictly normal. The diagnosis was based on a positive enterovirus RT-PCR on neonatal specimens: rectal, pharyngeal, and cord blood, as well as on maternal specimens. This article suggests that the diagnosis of fetal myocarditis due to enterovirus infection should be suspected in the presence of signs of cardiac failure associated with tachyarrhythmia [3].

Among the four EV infected patients in our prospective series, no ultrasound abnormalities were found in fetuses before delivery. Obviously, the low number of infected patients does not allow us to conclude.

There have been somewhat more postnatal than prenatal descriptions of congenital EV infection. In 1965, Moss and al. suggested for the first time the possibility of maternal-fetal transmission of EV [21]. Five fatal cases of neonatal infection with type 11 echovirus were reported in Boston area during the summer of 1979. The neonates presented with jaundice, hepatosplenomegaly, and liver failure leading to death. Virological diagnosis was performed by E-11 positive cultures in stool, urine and pharynx [22]. Piraino and al. reported similar findings with 13 cases of E-11 infection, 4 of which led to early neonatal death. These cases were described during the same summer of 1979 in Milwaukee [23]. Soudée and al. [5] studied the clinical, biological and demographic characteristics of newborns hospitalized for enterovirus infection in France in 2012. Data was retrieved from the database of the National Reference Center for Enterovirus Infections in Lyon. Among a total of 120 cases, 34 cases (28%) were diagnosed within the first 8 days of life. The clinical signs were respiratory and liver disease, including 11 severe cases (32%) defined by hepatitis (23%), myocarditis (12%), and encephalitis (15%) or multi-organ failure (32%) leading to death in 3 cases. Yen and al. reported the case of a premature born at 35 weeks deceased at 2 months of age from liver failure. The newborn presented shortly after birth with jaundice and altered consciousness. Five days before delivery by caesarean section, a foot-hand-mouth syndrome was reported in the mother. Echovirus-6 and 71 were found in newborn cultures (rectum, blood) [24]. Kao and al. reported a case of fatal neonatal Coxsackievirus B5 infection, confirmed by RT-PCR in the newborn and its mother [25]. The mother had fever for a week, 9 days before delivery, and a decrease in active fetal movements had been observed. The newborn developed fever immediately after birth and died on day one of life. This case is also suggestive of transplacental enterovirus transmission. Verboon-Maciolek and al reported six cases of severe enterovirus meningoencephalitis with severe white matter damage and clinical sequelae [6].

Although small numbers of patients have been described, based on the literature and taking into account the potential pathogenesis of enterovirus infections during pregnancy, we propose the following management.

In the case of a contact with an EV infected person, there is no need for a serological test, the result of which would be of no help. Basic hygiene measures should be explained and the patient should consult in case of fever in order to prevent fetal and/or neonatal complications.

We suggest that pregnant women presenting with a febrile syndrome without obvious bacterial cause, or confirmed influenza, should be offered enteroviruses RT-PCR, preferably on the mother’s plasma, possibly on an anorectal stool swab. However, a positive RT-PCR in the stool would reflect viral shedding and does not exclude other causes of maternal fever.

In case of confirmed maternal infection, close monitoring may be proposed.

Although there is no strategy evaluated in the literature, since the potential risks are cardiomyopathy, cerebral damage [18] and IUFD [17], we suggest fetal ultrasound follow-up on a monthly basis. In case of abnormal ultrasound findings during this follow-up, the frequency of ultrasound surveillance should be increased and an amniocentesis with RT-PCR for enteroviruses may be discussed. There is, however, no study to date establishing the diagnostic performance of RT-PCR for fetal EV.

At birth, if the mother has a proven infection with enteroviruses, testing for enterovirus should be offered with RT-PCR on cord blood. In the absence of newborn infection, there is no need for special surveillance. If the newborn is infected, considering the potential severity of enterovirus infections during the neonatal period, it is preferable to perform a liver function test, platelet count and hemostasis assessment as well as clinical monitoring [5].

## Conclusion

Enteroviruses are probably an underestimated cause of obstetrical and neonatal complications. Investigation of enterovirus by RT-PCR should be discussed during pregnancy and peripartum in case of febrile syndromes with no obvious bacterial cause, signs of fetal heart failure, or unexplained fetal death.

In case of positive EV testing after an intra-uterine fetal death, a diagnosis of EV infection will offer reassurance to parents on the lack of risk of recurrence in future pregnancies.
